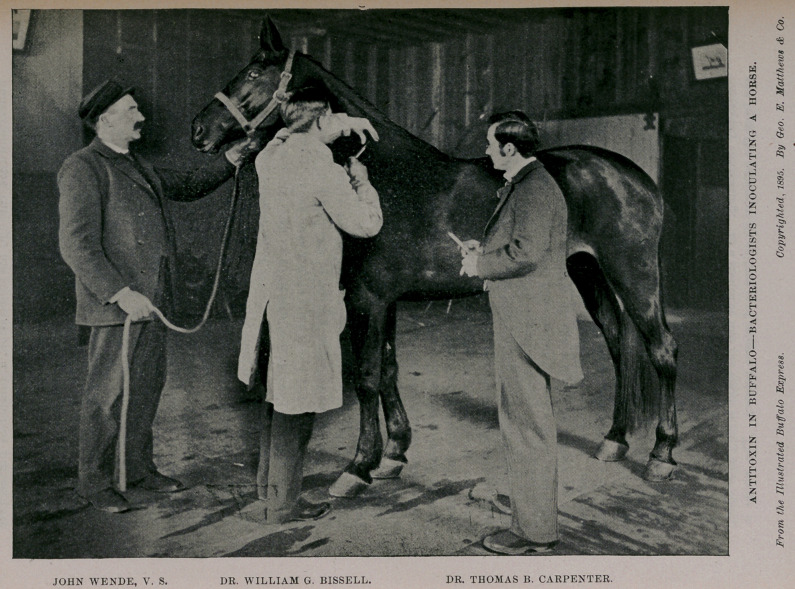# Antitoxin in Buffalo

**Published:** 1895-02

**Authors:** 


BUFFALO MEDICAL AND SURGICAL JOURNAL

A MONTHLY REVIEW OF MEDICINE AND SURGERY.

*EDITORS:*

THOMAS LOTHROP, M. D. - - WM. WARREN POTTER, M. D.

All communications, whether of a literary or business nature, should be addressed to the managing editor: 284 FRANKLIN STREET, BUFFALO, N. Y.

VOL. XXXIV. FEBRUARY, 1895. No. 7.

### ANTITOXIN IN BUFFALO.

Two physicians have recently fallen victims in this city of that dreaded and terribly destructive disease, diphtheria. The prominence of these professional men, and the circumstances under which they contracted the malady, lend additional sadness to their deaths. Both contracted the disease from patients whom they were serving, and each life was sacrificed while performing a noble service to humanity.

It is manifestly unfair to charge antitoxin with failure in cases where it has been employed at the eleventh hour and patients have died. During the illness of one of the physicians referred to, it is quite true that antitoxin was administered, but it is equally true, unless we are misinformed, that the first administration was made within fifteen hours of death. There is no reasonable probability that the remedy either hastened or delayed dissolution. On the other hand, there is every presumption that incurable toxemia existed at the very moment of its employment, and that no antidote, no matter how potent, could arrest its progress. It would be quite as sensible to expect success from the administration of an antidote to any irritant or lethal poison after it had permeated all the "natural gates and alleys of the body."

In view of the recent investigations with reference to the serum treatment of diphtheria, it is difficult to escape the conviction that both of these lives might have been saved had the antitoxin treatment been adopted early in their progress. No stronger argument can be made for the manufacture of the blood serum by local enterprise than is presented in the deaths of these two prominent young physicians.

We are pleased to observe that steps have been taken looking to its cultivation by the health commissioner, Dr. Wende. We

JOHN WENDE, V. S. DR. WILLIAM G. BISSELL. DR. THOMAS B. CARPENTER.

ANTITOXIN IN BUFFALO—BACTERIOLOGISTS INOCULATING A HORSE.

*From the Illustrated Buffalo Express.* *Copyrighted, 1895. By Geo. E. Matthews & Co.*

understand that, through his solicitation, a number of horses have been supplied and inoculations have already been made. These steps have been taken without expense to the city, the necessary funds and animals sufficient to make a start, having been furnished through private contribution.

Other cities have not hesitated to appropriate large sums of money for the purpose of carrying on the manufacture of antitoxin, and if further investigation should support the already strongly rooted belief in the preventive efficacy of the blood serum, it is difficult to see how Buffalo can refuse to do the same. It seems to be pretty well established already that its effectiveness is in direct ratio to its early employment, and it is hard to ask a humane profession to stand helplessly at the bedside of dying patients deprived of the one remedy, that, of all others, seems to offer the most certain relief.

If this remedy is to be successfully employed at all, it must be applied early, as we have before stated, and, in order to use it sufficiently early in diphtheria to warrant expectation of cure, a supply must be constantly accessible to all physicians who are called upon to treat this disease. We are aware that antitoxin has not yet passed its experimental stage, but results are already sufficiently promising to justify a further vigorous prosecution of these experiments on the highest plane of scientific work. In the further pursuit of these experiments we do not feel willing that the City of Buffalo shall stand idly by and not contribute her full share to the general result, whatever it may finally prove to be.

In interesting relationship to this subject we publish herewith a picture showing the method of inoculating horses with diphtheria serum, in operation by the health department.

In the *Buffalo Illustrated Express* for January 20th was published an excellent description of the method of obtaining the toxin, and the manner in which animals are rendered immune. We reproduce it here as a matter of current interest:

The toxin with which the animal is inoculated is secured by cultivating the diphtheria germs. The bacteria are gathered from a particularly virulent case of diphtheria, and are planted in fluid bouillon, contained in a glass dish of peculiar shape, which is kept at a temperature of about 37 centigrades of heat (equal to 98 or 98½ F., or the normal temperature of the body), and over which a current of air is constantly circulating. In a month or less the toxin is fully deve'oped, and from the nature of its culture is of intense potency. The whole is then put in a Pasteur-Chamberlin filter, and the bacteria are separated from the bouillon culture. The strength of the toxin in the filtrate is next ascertained by testing it upon some animal of known susceptibility, usually a guinea-pig. The test is made by finding how much of it will kill an animal of given weight in a given time. It has been demonstrated that one-tenth of a cubic centimeter of bouillon-cultured toxin will kill a guinea-pig weighing 500 grammes in forty-eight hours. Having ascertained the precise strength of the toxin, the next move is to grow the antitoxin, and for this purpose a healthy animal, preferably a horse, not less than five nor more than ten years old, is inoculated with the diphtheritic poison. The first dose is a half to one cubic centimeter (about one-third of a teaspoonful), injected subcutaneously in the horse's neck, and repeated in constantly-increasing doses at intervals of five or ten days, according to the effect upon the animal. The process converts a strong horse into a sick one, and inoculation and subsequent care and bleeding should be under the superintendence of a competent veterinarian. At the points of injection there is considerable swelling and more or less edema, the animal refuses food, has a heightened temperature and acts generally like a very sick horse. As soon as the temperature is reduced and the animal begins to recover, more toxin is injected. Too much at any time would kill him, but by a system of gradual increase the last dose may be 300 or 400 times stronger than the first, when he is ready to yield his blood for the serum that is in it. The time occupied in the complete immunization of the horse from the disease varies from four to nine or ten months. The average is about six. His blood is tested, and when it is found to possess strong antitoxic power, the jugular vein is tapped and as much blood withdrawn as the veterinarian deems safe. Then, by a simple method, the efficiency of the serum of the blood is carefully and accurately determined, when it is ready for distribution, so many centimeters to so many pounds of diphtheritic patient, for the antitoxin dosage is so computed.

## Figures and Tables

**Figure f1:**